# Relationship Between the Levels of Feno, Blood Eosinophils, and the Severity of Exacerbations in Patients With COPD: Results From COSYCONET

**DOI:** 10.1016/j.chpulm.2025.100189

**Published:** 2025-06-20

**Authors:** Peter Alter, Henrik Watz, Kathrin Kahnert, Franziska C. Trudzinski, Hubert Wirtz, Tim Speicher, Inge Kokot, Sandra Söhler, Robert Bals, Klaus F. Rabe, Emiel F.M. Wouters, Claus F. Vogelmeier, Rudolf A. Jörres

**Affiliations:** a Department of Medicine, Pulmonary, Critical Care and Sleep Medicine, Philipps University of Marburg (UMR), German Center for Lung Research (DZL), Marburg, Germany; b Velocity Clinical Research Germany GmbH, Ahrensburg, Germany; c Department of Internal Medicine V, University Hospital, LMU Munich, Comprehensive Pneumology Center Munich (CPC-M), German Center for Lung Research (DZL), Munich, Germany; d MediCenterGermering, Germering, Germany; e Department of Pneumology and Critical Care Medicine, Thoraxklinik, University of Heidelberg, Translational Lung Research Center Heidelberg (TLRC-H), Center for Lung Research (DZL), Heidelberg, Germany; f Department of Internal Medicine I, Pneumology, University of Leipzig, Leipzig, Germany; g Department of Internal Medicine V - Pulmonology, Allergology, Intensive Care Medicine, Saarland University Hospital, Homburg, Germany; h LungenClinic Grosshansdorf and Department of Medicine, Christian-Albrechts University, Kiel, Airway Research Center North (ARCN), German Center for Lung Research (DZL), Grosshansdorf, Germany; i Department of Respiratory Medicine, Maastricht University Medical Centre, Maastricht, The Netherlands; j Sigmund Freud Private University, Medical Faculty, Vienna, Austria; k Institute and Outpatient Clinic for Occupational, Social and Environmental Medicine, University Hospital, LMU Munich, Comprehensive Pneumology Center Munich (CPC-M), German Center for Lung Research (DZL), Munich, Germany

**Keywords:** COPD, eosinophils, exacerbation, exhaled nitric oxide (FeNO)

## Abstract

**Background:**

Exacerbation risk of patients with COPD is thought to be influenced by type 2 inflammation, with blood eosinophil counts and fractional concentration of exhaled nitric oxide (Feno) as potential biomarkers.

**Research Question:**

Are there different associations of blood eosinophils and Feno with exacerbation risk and severity in COPD, indicating a different role of local inflammation vs systemic factors?

**Study Design and Methods:**

Data were taken from 3 visits (1.5 years apart) of the longitudinal COPD and Systemic Consequences–Comorbidities Network (COSYCONET) cohort, comprising a broad range of clinical and functional assessments. We determined the relationships between eosinophil counts and Feno vs exacerbations, defined either via categorization to Global Initiative for Chronic Obstructive Lung Disease group E (≥ 2 moderate or ≥ 1 severe), or as ≥ 1 severe exacerbation in the year before each visit. Analyses were performed via generalized linear models.

**Results:**

The final data set included 384, 255, and 206 patients at visits 6, 7, and 8, respectively. According to the multivariable analyses, exacerbation risk defined via Global Initiative for Chronic Obstructive Lung Disease group E was associated with elevated values (≥ 25 ppb) of Feno (*P* = .003; OR, 1.90), but not eosinophil counts. In contrast, the risk for severe exacerbations was linked to eosinophils, but not to Feno. This relationship was expressed as either elevated risk with counts ≥ 100 and < 300 M/L (*P* = .017; OR, 1.98) or as reduced risk (*P* = .046; OR, 0.57) < 100 M/L. The results were robust against the inclusion of patients who actively smoke or with the comorbidity of asthma.

**Interpretation:**

Our observations suggest a differential role of type 2-related biomarkers Feno and eosinophils for exacerbation risk and severity. Feno seemed superior regarding a broad range of exacerbations predominantly involving local airway events, whereas systemic eosinophils played a larger role in severe exacerbations. The findings also suggest that a low eosinophil count might indicate a low risk of severe exacerbations, whereas highly elevated counts did not play a statistical role.

**Clinical Trial Registration:**

ClinicalTrials.gov; No.: NCT01245933; URL: www.clinicaltrials.gov

## Graphical Abstract

**Figure:**
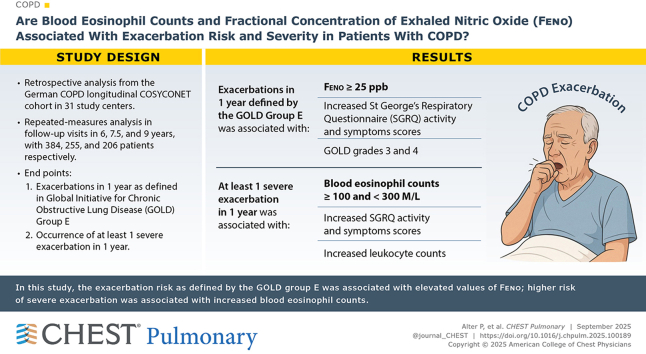

**Take-Home Points**

**Study Question:** Are blood eosinophil counts and fractional concentration of exhaled nitric oxide (Feno) associated with exacerbation risk and severity in patients with COPD?

**Results:** Exacerbation risk as defined in terms of Global Initiative for Chronic Obstructive Lung Disease group E allocation was associated with elevated Feno concentration, whereas the risk for severe exacerbation was linked to blood eosinophils.

**Interpretation:** Findings suggest a differential role of type 2-related inflammation as indicated by the biomarkers Feno and eosinophils for COPD exacerbation risk and severity.

Exacerbations are considered important adverse events in patients with COPD.^1^, ^2^, ^3^ Studies have revealed that type 2 inflammation plays a role not only in asthma,^4^ but also in COPD, and is linked to increased lung function decline.^5^ Elevated eosinophil counts have been associated with an increased risk for readmission after exacerbation,^6^ and COPD subgroups reveal a link between eosinophilic inflammation and the risk or severity of exacerbations.^5^, ^6^, ^7^ Based on this, novel treatment strategies^4^^,^^7^ use compounds capable of attenuating eosinophilic inflammation, specifically biologicals targeting various cytokines. For this purpose, antibodies against IL-13, IL-4, IL-5, and thymic stromal lymphopoietin have been studied. Results were partially equivocal,^8^^,^^9^ but favorable effects on the frequency of moderate and severe exacerbations were found.^10^, ^11^, ^12^ Usually, eosinophil counts in peripheral blood are considered as an easily accessible marker of the propensity to respond to this kind of treatment, with different clinical cutoff values, similar to those proposed for asthma.^13^

It is also known that type 2 inflammation of the respiratory tract is linked to elevated values of fractional concentration of exhaled nitric oxide (Feno).^14^^,^^15^ Therefore, in principle, Feno could also serve as an indicator of eosinophil-related exacerbation risk.^16^ Indeed, several studies found this type of association,^17^^,^^18^ in addition to that with blood eosinophil counts.^19^^,^^20^ Feno has even been reported to be superior to eosinophil counts regarding its link to exacerbation risk,^21^ in accordance with data on the absence of a significant relationship between Feno and blood eosinophils.^17^ However, even a lower frequency of moderate and severe exacerbations in patients with elevated values of Feno has been described.^22^ On the 1 hand, these results underline the complexity of COPD pathophysiology; on the other hand, they raise the question whether the associations depend on the severity of exacerbations.

It might be argued, for example, that Feno more closely reflects local conditions within the respiratory tract than blood eosinophils that might be more indicative of systemic alterations. Based on this, one might further assume that Feno better matches the overall risk for exacerbations, including those focused on the airways, whereas blood eosinophil counts better match the risk for severe exacerbations that are more likely to include a systemic component. To test this hypothesis, we investigated whether the association between blood eosinophil counts, Feno, and exacerbations depends on exacerbation severity. For this purpose, we first took the definition of Global Initiative for Chronic Obstructive Lung Disease (GOLD) group E^23^ to signify the occurrence of at least ≥ 2 moderate or ≥ 1 severe exacerbation per year; we second had the explicit requirement of at least 1 severe exacerbation. The dataset used for the analysis was that of the longitudinal COPD and Systemic Consequences–Comorbidities Network (COSYCONET) COPD cohort.^24^

## Study Design and Methods

### Study Population

Patients of the German multicenter COSYCONET cohort were included. The cohort included patients with postbronchodilator spirometric COPD grades 1 to 4 according to GOLD,^23^^,^^25^ and patients of the former GOLD grade 0^26^^,^^27^ (ie, individuals with chronic bronchitis who did not fulfill the criterion of the ratio of FEV_1_ to FVC < 0.7). The study protocol, inclusion and exclusion criteria, and panel of assessments performed at each study visit have been published previously.^24^ The present analysis was based on data from visits 6, 7, and 8, which took place 6, 7.5, and 9 years, respectively, after the recruitment visit. This selection was because only at these visits were blood eosinophil counts and values of Feno available in numbers sufficient for analysis. All visits were subject to the requirement that patients were in a stable clinical condition and potential previous exacerbations had occurred at least 4 weeks prior. The study was approved by the ethics committees of all participating study centers, and was performed according to the revised Declaration of Helsinki. All participants gave their written informed consent.

### Assessments

Anthropometric information including height, weight, and smoking status (active) was obtained using standard procedures^24^; from height and weight, BMI was computed. Forced spirometry after inhalation of bronchodilators^24^ was performed according to recommendations.^28^^,^^29^ It comprised the determination of FEV_1_, FVC, and the ratio FEV_1_/FVC. To evaluate the results, predicted values of the Global Lung Function Initiative were used.^30^^,^^31^

The modified Medical Research Council dyspnea score^32^ was used for the categorization of patients into GOLD groups A/B/E,^23^ whereby exacerbation risk was evaluated as proposed by GOLD and used in previous analyses of COSYCONET data.^33^, ^34^, ^35^, ^36^ A positive history according to group E was assumed with at least 2 moderate exacerbations or at least 1 severe exacerbation in the previous year, and the occurrence of severe exacerbations was also recorded separately. Health-related quality of life was assessed with the St. George’s Respiratory questionnaire (SGRQ) with its 3 subscores addressing activity, symptoms, and impact.^37^ Generic quality of life was determined with the EuroQoL-5-dimension questionnaire, using its visual analog scale (VAS) in the present analysis. We did not include data from the COPD Assessment Test because of its high redundancy with the other questionnaires in the multivariable analyses.

Blood eosinophil counts (absolute values, M/L) were determined from samples acquired at each study visit and analyzed in the local laboratories of the study centers. Correspondingly, the values of Feno (ppb) were derived from measurements at a standard expiratory flow rate of 50 mL/s,^38^ as average of 2 valid measurements. As measuring devices, the study centers used the Aerocrine Niox (737 assessments), Bedfont NObreath (15 assessments), Ecomedics Analyzer CLD 88 sp (19 assessments), and Medisoft Feno + (72 assessments); no information was available for 2 measurements. These devices are widely distributed in clinical practice, and there is no hint that they show systematic differences of relevant magnitude in their measured values.

### Statistical Analysis

For data description, numbers and percentages were computed, and mean values and SDs. In case of Feno and eosinophil counts, values were logarithmically transformed to achieve a more symmetrical data distribution. Accordingly, geometric mean values and SDs were computed for these 2 variables, with geometric SDs to be interpreted as variability factors. For blood eosinophil counts, we also defined a categorical variable with 3 values indicating whether counts were < 100 M/L, ≥ 100 and < 300 M/L, or ≥ 300 M/L; for Feno, we had a binary variable indicating whether values were ≥ 25 ppb. Comparisons were performed by generalized linear models (GLMs) using the repeated measurements design for corresponding variables, with a logit link for the binary outcomes at the respective visit of either being categorized as GOLD group E, or of at least 1 severe exacerbation in the previous year. In the analyses, major anthropometric and clinical characteristics that could be reasonably linked to exacerbation risk were included as covariates. Statistical significance was assumed at *P* < .05, and all analyses were performed using the SPSS software package (Version 29; IBM).

## Results

### Patient Description

At visit 6, 776 patients participated in COSYCONET, whereas at visits 7 and 8, 492 and 375 patients, respectively, took part in the assessments. Of these patients, 714, 439, and 321 had complete data (as required for Table 1, except Feno) including eosinophil counts at visits 6, 7, and 8, respectively. When additionally requiring Feno measurements, the respective numbers of patients were reduced to 384, 255, and 206, originating from 14 study centers. These patients comprised the final study population, and their characteristics at visits 6, 7, and 8 are given in Table 1. There was a trend over time toward lower prevalence of GOLD group E and of severe exacerbations, but this was not statistically significant in the multivariable analyses (subsequently discussed).

**Table 1:** Characteristics of the Patients at Visits 6, 7, and 8

Variable	Visit 6 (n = 384)	Visit 7 (n = 255)	Visit 8 (n = 206)
Sex, M/F	231 (60.2)/153 (39.8)	151 (59.2)/104 (40.8)	123 (59.7)/83 (40.3)
Age, y	69.5 [8.0]	70.7 [8.0]	71.5 [8.1]
BMI, kg/m^2^	26.7 [5.0]	26.9 [5.3]	27.3 [5.1]
BMI < 20 kg/m^2^	30 (7.8)	25 (9.8)	20 (9.7)
BMI ≥ 30 kg/m^2^	93 (24.2)	65 (25.5)	60 (29.1)
Smoking (current)	61 (15.9)	41 (16.1)	30 (14.6)
FEV_1_ % predicted	57.9 [21.3]	59.5 [21.4]	61.8 [21.9]
FVC % predicted	82.8 [19.2]	83.1 [20.0]	84.5 [19.9]
GOLD grade 0/1/2/3/4	43 (11.2)/40 (10.4)/143 (37.2)/128 (33.3)/30 (7.8)	30 (11.8)/30 (11.8)/106 (41.6)/70 (27.5)/19 (7.5)	31 (15.0)/27 (13.1)/78 (37.9)/61 (29.6)/9 (4.4)
GOLD group A/B/E	180 (46.9)/106 (27.6)/98 (25.5)	124 (48.6)/78 (30.6)/53 (20.8)	100 (48.5)/65 (31.6)/41 (19.9)
Severe exacerbation	49 (12.8)	28 (11.0)	19 (9.2)
SGRG total	38.7 [19.7]	36.9 [19.6]	38.9 [20.3]
SGRQ activity	54.7 [26.3]	52.7 [27.8]	56.0 [28.7]
SGRQ impact	25.3 [20.3]	24.1 [18.8]	25.0 [19.3]
SGRQ symptoms	50.1 [21.4]	46.9 [21.2]	50.0 [22.4]
EQ-5D-VAS	58.3 [19.1]	59.2 [19.0]	58.2 [20.6]
Leukocytes, G/L	7.7 [2.5]	7.8 [2.3]	7.7 [2.1]
Eos, M/L^a^	153.1 [2.6]	144.9 [2.6]	144.2 [2.6]
Eos < 100, 100-300, ≥ 300 M/L	91 (23.7)/216 (56.3)/77 (20.1)	68 (26.7)/134 (52.5)/53 (20.8)	49 (23.8)/118 (57.3)/39 (18.9)
Feno, ppb^a^	15.0 [2.0]	15.2 [2.0]	15.9 [1.9]
Feno ≥ 15 ppb	183 (47.7)	129 (50.6)	115 (55.8)
Feno ≥ 25 ppb	86 (22.4)	56 (22.0)	44 (21.4)

Data are presented as absolute No. (%), arithmetic mean [SD], or as otherwise indicated. Eos = blood eosinophils, EQ-5D-VAS = visual analog scale of the European Quality of Life 5 Dimensions Questionnaire; F = female; Feno = fractional concentration of exhaled nitric oxide; GOLD = Global Initiative for Chronic Obstructive Lung Disease; M = male; SGRQ = St. George’s Respiratory questionnaire.

a Geometric mean [SD]. Note that geometric SDs are to be interpreted as variability factors and not as additive terms.

### Association Between Exacerbations and Single Markers

Univariate analyses taking into account the repeated measurements via GLM, revealed that there were no significant associations between GOLD group E and sex, age, BMI (either continuous, or 3 categories: low, medium, or high, defined via cutoff values of 20 and 30 kg/m^3^), active smoking, blood eosinophils (either continuous with log10-transformed values, or 3 categories), SGRQ impact score, or GOLD grades 1 and 2. There were, however, associations with GOLD grades 3 and 4 (either separate or combined, *P* < .001 each), SGRQ activity and symptoms scores (*P* < .001 each), VAS (*P* < .001), blood leukocytes (*P* = .004), log10 of Feno (*P* = .002), and the binary category of Feno ≥ 25 ppb (*P* < .001).

Regarding severe exacerbations, only GOLD grades 3 and 4 (either separate or combined, *P* < .001 each), SGRQ activity and symptoms scores (*P* ≤ .002 each), VAS (*P* < .001), blood leukocytes (*P* < .001), and eosinophils ≥ 100 and < 300 M/L (*P* = .028) showed statistically significant associations. When testing the lowest category < 100 M/L against its complement, there was a borderline significant dependence (*P* = .059, Fisher exact text).

**Figure 1:**
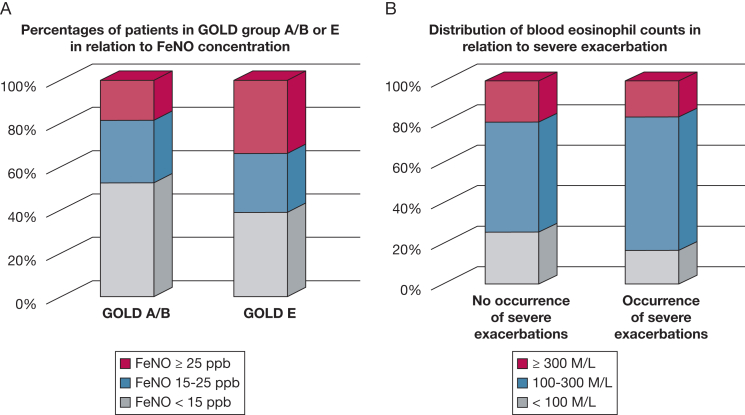
A, Percentages of patients in either GOLD group A/B or group E (elevated exacerbation history) showing Feno values either < 15 ppb, or of at least 25 ppb, or in between 15 and 25 ppb. There was a statistically significant association with exacerbations (P < .001). B, Percentages of patients with the 3 categories of blood eosinophil counts (<100, ≥ 100 to < 300, ≥ 300 M/L) among those in whom either no severe exacerbations occurred, or severe exacerbations occurred. Feno = fractional concentration of exhaled nitric oxide; GOLD = Global Initiative for Chronic Obstructive Lung Disease.

The relationships between GOLD group E and different Feno ranges and between severe exacerbations and different eosinophil ranges are illustrated in Figure 1.

### Multiple Risk Factors of Exacerbations in Terms of GOLD Group E

To account for the simultaneous relationship of predictors to outcome variables, we used the GLM with a number of simultaneous predictors. The results are shown in Table 2. Significant predictors (*P* < .05 each) were GOLD grades 3 and 4, SGRQ activity and symptoms scores, and Feno ≥ 25 ppb (*P* < .003). There was, however, no association of this type of exacerbations with blood eosinophil counts.

**Table 2:** Results of the Longitudinal Logistic Regression Analysis With the History of Exacerbations in 1 Year as Defined in GOLD Group E (≥ 1 Severe or ≥ 2 Moderate) as the Dependent Variable, Based on Data From Visits 6, 7, and 8

Variable	Regression Coefficient		OR			*P* Value
	Estimate	SEM	Estimate	Lower 95% CI Limit	Upper 95% CI Limit	
Visit 8	−0.337	0.216	0.714	0.467	1.090	.119
Visit 7	−0.130	0.205	0.878	0.588	1.311	.524
Sex, female	0.330	0.221	1.391	0.901	2.147	.136
Age per 10 y	−0.084	0.141	0.920	0.697	1.214	.555
BMI < 20 kg/m^2^	−0.015	0.326	0.985	0.519	1.867	.963
BMI ≥ 30 kg/m^2^	−0.130	0.242	0.878	0.546	1.412	.592
Smoking active	−0.398	0.323	0.672	0.357	1.265	.218
GOLD grade 3/4 vs 0/1/2	0.563	0.213	1.757	1.158	2.665	**.008**
SGRQ activity per 10 score points	0.172	0.062	1.188	1.051	1.343	**.006**
SGRQ impact per 10 score points	−0.022	0.077	0.978	0.841	1.138	.774
SGRQ symptoms per 10 score points	0.309	0.061	1.362	1.210	1.534	**< .001**
EQ-5D-VAS per 10 of 100 mm	−0.054	0.062	0.948	0.839	1.071	.389
Leukocytes, G/L	0.066	0.044	1.068	0.980	1.165	.135
Blood eosinophils ≥ 300 M/L	0.408	0.301	1.504	0.834	2.712	.175
Blood eosinophils ≥ 100 and < 300 M/L	0.336	0.243	1.399	0.868	2.254	.168
Feno ≥ 25 ppb	0.640	0.219	1.896	1.235	2.911	**.003**

Regression coefficients, corresponding ORs (= exp (coefficient)), their 95% CIs, and *P* values for each coefficient are shown. Statistically significant predictors are marked in boldface font. The corrected quasi-likelihood of the model regarding the independence criterion (QICC) was 760.481, indicating a slightly improved overall fit when compared with 768.460 for a model without eosinophils and Feno. EQ-5D-VAS = visual analog scale of the European Quality of Life 5 Dimensions Questionnaire; Feno = fractional concentration of exhaled nitric oxide; GOLD = Global Initiative for Chronic Obstructive Lung Disease; SGRQ = St. George’s Respiratory questionnaire.

### Multiple Risk Factors of Severe Exacerbations

The analysis was performed in the same way as for GOLD group E, and the results are shown in Table 3. The SGRQ activity and symptoms scores were significantly (*P* < .05 each) linked to severe exacerbations, and leukocyte counts and the counts of blood eosinophils being ≥ 100 and < 300 M/L. On the contrary, there was no significant dependence of Feno with any of the cutoff values.

**Table 3:** Results of the Longitudinal Logistic Regression Analysis With the Occurrence of At Least 1 Severe Exacerbation in 1 Year as the Dependent Variable, Based on Data From Visits 6, 7, and 8

Variable	Regression Coefficient		OR			*P* Value
	Estimate	SEM	Estimate	Lower 95% CI Limit	Upper 95% CI Limit	
Visit 8	−0.484	0.306	0.616	0.338	1.122	.113
Visit 7	−0.054	0.269	0.948	0.559	1.606	.842
Sex, female	−0.034	0.283	0.967	0.555	1.683	.906
Age per 10 y	0.079	0.193	1.082	0.741	1.579	.684
BMI < 20 kg/m^2^	−0.601	0.541	0.548	0.190	1.583	.266
BMI ≥ 30 kg/m^2^	−0.458	0.295	0.632	0.355	1.127	.120
Smoking active	−0.429	0.416	0.651	0.288	1.474	.303
GOLD grade 3/4 vs 0/1/2	0.514	0.303	1.673	0.923	3.030	.090
SGRQ activity per 10 score points	0.365	0.081	1.441	1.228	1.690	**< .001**
SGRQ impact per 10 score points	−0.170	0.096	0.844	0.699	1.018	.076
SGRQ symptoms per 10 score points	0.236	0.078	1.266	1.086	1.476	**.003**
EQ-5D-VAS per 10 of 100 mm	0.041	0.072	1.042	0.905	1.200	.566
Leukocytes, G/L	0.148	0.049	1.159	1.053	1.276	**.003**
Blood eosinophils ≥ 300 M/L	0.137	0.410	1.147	0.513	2.565	.738
Blood eosinophils ≥ 100 and < 300 M/L	0.685	0.287	1.984	1.130	3.484	**.017**
Feno ≥ 25 ppb	0.356	0.280	1.428	0.824	2.473	.204

Regression coefficients, corresponding ORs (= exp (coefficient)), their 95% CIs, and *P* values for each coefficient are shown. Statistically significant predictors are marked in boldface font. The corrected quasi-likelihood of the model regarding the independence criterion was 520.763, indicating a very slight improvement of fit compared with 522.779 for a model without Feno and eosinophils. EQ-5D-VAS = visual analog scale of the European Quality of Life 5 Dimensions Questionnaire; Feno = fractional concentration of exhaled nitric oxide; GOLD = Global Initiative for Chronic Obstructive Lung Disease; SGRQ = St. George’s Respiratory questionnaire.

### Sensitivity Analyses

Because it is possible that the predictive value of eosinophil counts and Feno depends on the presence of the comorbidity asthma, we repeated the analysis while including the patient-reported physician-based diagnosis of asthma, which was recorded in 23.4% of patients. This did not alter the pattern of significant associations and left the respective regression coefficients (ORs) essentially unaffected. Moreover, we compared various cutoff values for Feno because a traditional receiver operating characteristics analysis was not feasible because of the repeated-measures design. Regarding the association with GOLD group E, similar results for Feno ≥ 25 ppb but with a lower level of significance were obtained, when using cutoff values of 20 ppb (*P* = .023) or 15 ppb (*P* = .011) for Feno, whereas regarding severe exacerbations, the lack of association with Feno remained. Thus, the association of exacerbations in terms of GOLD group E was fairly robust against the choice of the specific cutoff value of Feno.

The category GOLD group E included both moderate and severe exacerbations, thereby showing some overlap with the category of severe exacerbations; it was chosen primarily as a convenient and well-introduced definition. When repeating the analysis with a category comprising only moderate exacerbations as defined in GOLD group E but excluding severe exacerbations, essentially the same results regarding Feno and eosinophils were obtained. Moreover, the pattern of significant associations with Feno and eosinophils remained the same when excluding patients who currently smoke from the analysis. When simplifying the 3 categories of eosinophils by a binary category of eosinophils being < 100 M/L, this was statistically significant (*P* = .046) for severe exacerbations, with an OR of 0.57. This result emphasized a lower risk for severe events with low eosinophil numbers, but no role for elevated numbers.

## Discussion

In the present work, we found that an elevated COPD exacerbation risk as defined via GOLD group E was associated with elevated Feno values but not with blood eosinophil counts, indicating the diagnostic superiority of Feno in this regard. The critical level of Feno was 25 ppb, and the likelihood of belonging to GOLD group E was about twice as high as in the case when Feno was < 25 ppb. GOLD group E requires at least 2 moderate or at least 1 severe exacerbation in the previous year; thus, it is not homogeneous. We then hypothesized that severe exacerbations would be more closely linked to eosinophils compared with Feno, and this was confirmed. Noteworthy enough, the range relevant for elevated risk was 100 to 300 M/L, and there was no increasing risk with values > 300 M/L, which is often used in therapeutic decisions.^7^^,^^11^^,^^12^ Conversely, the results could be interpreted in the way that eosinophil counts < 100 M/L were relevant, indicating a decrease in the risk for severe exacerbations by nearly factor 2. In view of the known variability of high eosinophil counts,^39^^,^^40^ these observations seem interesting. They naturally raise the question of whether it would be more adequate to exclude patients from type 2-targeted therapies via low eosinophil counts than to include them via high eosinophil counts. Our study was not designed to address this question, but the results might lead to further considerations and possible retrospective analyses of available data.

Because eosinophils and Feno are both related to type 2 inflammation, which is particularly important in asthma, it was natural to ask whether the presence of asthma as a comorbidity affected the results. About 23% of patients reported this comorbidity, but the results in terms of Feno, eosinophils, and their associations with GOLD group E or severe exacerbations remained essentially the same if asthma was introduced as a predictor into the analyses or these patients were excluded. In our presentation, we preferred to exclude asthma as a predictor because of the known difficulties encountered in real clinical practice when determining the presence of this comorbidity.

In a similar manner, the specific cutoff value for Feno was not essential for its different relationship to GOLD group E vs severe exacerbations. A comparison of cutoff values revealed that 25 ppb showed the most significant association with GOLD group E, irrespective of the inclusion of patients who actively smoke, which could affect the measurement of Feno.^41^ In conclusion, the role of Feno regarding GOLD group E was not hampered by the inclusion of patients who actively smoke, which was about 15% in the present study population, and the relationship between Feno and GOLD group E was therefore fairly robust.

This is relevant because among the easily accessible markers of airway inflammation, undoubtedly Feno is the most feasible tool. The use of spontaneous sputum, for example, heavily depends on sample quality,^42^ and that of induced sputum requires expertise, time, and resources that are not normally available in clinical practice. Moreover, the use of bronchial biopsies and bronchoalveolar lavage fluid is limited to dedicated studies and completely unrealistic for broad-scale routine purposes.

Regarding severe exacerbations but not GOLD group E, we also found a significant role of leukocyte counts. It is undisputable that leukocytes play a role for the course of the disease and exacerbations, as shown in many studies and demonstrated for mortality in COSYCONET data.^43^ This association is plausible because it can be assumed that particularly severe exacerbations are associated with mortality; thus, leukocyte counts are linked to both of them. The findings on overall leukocyte counts also match the observation that eosinophil counts were associated with severe exacerbations only, but not with the more general exacerbation risk as defined via GOLD group E.

The comparison of eosinophil counts vs Feno is probably related to the distinction between local and systemic inflammation. This was the starting point of our analyses comprising eosinophils and Feno on the one hand, and GOLD group E and severe exacerbations on the other hand. It would also have been of interest to include mild exacerbations; however, the number of these exacerbations as reported in COSYCONET was low and raised the suspicion that either such exacerbations were rare or that they were underreported. In any case, our findings demonstrated that the distinction between moderate to severe and severe exacerbations was already sufficient to reveal differential roles for an airway marker and a systemic marker. The dominant role of systemic effects in severe exacerbations was also supported by the observation that their occurrence did not depend on spirometric GOLD grades, specifically GOLD grades 3 and 4, in contrast with the exacerbations defined via GOLD group E. These explanations remain speculative but seem plausible and could be the starting point for more detailed analyses.

Although generic quality of life as quantified by the EuroQoL-5-dimension questionnaire (EQ-5D) visual analog scale (VAS) was no longer associated with exacerbations in the multivariable analyses, the SGRQ activity and symptoms scores remained significant compared with the univariate analyses. Interestingly, the SGRQ impact score was not correlated with exacerbations, possibly because this score has some dependence on questions that are prone to adaptation of the patients’ perception to their impairments, in contrast with the more objective questions defining the SGRQ activity and symptoms scores. This underlines the clinical usefulness of the SGRQ, particularly in the absence of functional data and the relevance of distinguishing between its subscores.

An obvious limitation of our results is that they are descriptive in nature because of the observational design of the analyses. Despite this, they might add to the current discussion on the usefulness of biomarkers for treatment decisions, particularly regarding compounds that address type 2 inflammation.^4^^,^^11^^,^^12^^,^^44^ Our results are in line with previous findings that Feno may play a role in such decisions.^5^^,^^15^^,^^40^^,^^45^ They also support the role of blood eosinophil counts that is broadly accepted but at the same time suggest that the role of high counts^46^ might be reconsidered. It is well known that eosinophil counts are rather variable,^47^, ^48^, ^49^ and this variability has also been demonstrated for COSYCONET patients.^50^ As suggested by our data, a potential alternative could be to use the presence of low eosinophil counts (< 100 M/L) to conclude that the risk of severe exacerbations is reduced and therefore compounds addressing such exacerbations via systemic type 2 inflammation might be less effective in these patients. At present, however, this only remains an intriguing speculation that must be further tested. A potential objection to our results could be that we included all patients with complete data at a specific visit but with potentially < 3 visits. Because of the reduction in case numbers, it was not possible to perform an alternative analysis using only patients with 3 study visits. However, we consider it unlikely that this had an effect because the major selective loss of patients in COSYCONET appears to have occurred over the first few visits. Moreover, it is not likely that the use of different (validated) Feno analyzers by different study centers affected the results because the distribution of Feno values was not associated with these devices.

## Interpretation

A retrospective analysis of data from a COPD cohort showed that the generic exacerbation risk as defined via GOLD group E correlated with elevated values of Feno, but not with blood eosinophil counts. In contrast, the risk for severe exacerbations correlated with blood eosinophil counts, but not with Feno. This correlation was expressed in the fact that lower counts were associated with a lower risk of severe exacerbations, whereas highly elevated counts did not play a statistical role. These findings are intriguing but need to be verified in further studies.

## Funding/Support

COSYCONET is supported by the 10.13039/501100002347German Federal Ministry of Education and Research (BMBF) Competence Network Asthma and COPD (ASCONET) and performed in collaboration with the 10.13039/501100010564German Center for Lung Research (DZL). The project is funded by the BMBF [Grant 01 GI 0881] and DZL [Grant 82DZLI05B2]. It is also supported by unrestricted grants from AstraZeneca GmbH, Bayer Schering Pharma AG, Boehringer Ingelheim Pharma GmbH & Co KG, Chiesi GmbH, GlaxoSmithKline, Grifols Deutschland GmbH, MSD Sharp & Dohme GmbH, Mundipharma GmbH, Novartis Deutschland GmbH, Pfizer Pharma GmbH, Sanofi-Aventis Deutschland GmbH, Takeda Pharma Vertrieb GmbH & Co KG, and Teva GmbH for patient investigations and laboratory measurements.

## Financial/Nonfinancial Disclosures

None declared.
